# Supplementary material for: FDA and EMA Oversight of Disruptive Science on Application of Finite Absorption Time (F.A.T.) Concept in Oral Drug Absorption: Time for Scientific and Regulatory Changes
Source: Pharmaceutics. 2024 Nov 11;16(11):1435. doi: 10.3390/pharmaceutics16111435 (PMC11597828; doi:10.3390/pharmaceutics16111435)
Supplement: Supplementary file 1 [file pharmaceutics-16-01435-s001.zip › pharmaceutics-3287766-supplementary.pdf]

FDA and EMA oversight of disruptive science on the application of Finite Absorption Time (F.A.T.) concept in oral drug absorption: Time for scientific and regulatory changes

E. Toulitsis, A. A. Tsekouras, P. Macheras

Supplementary information

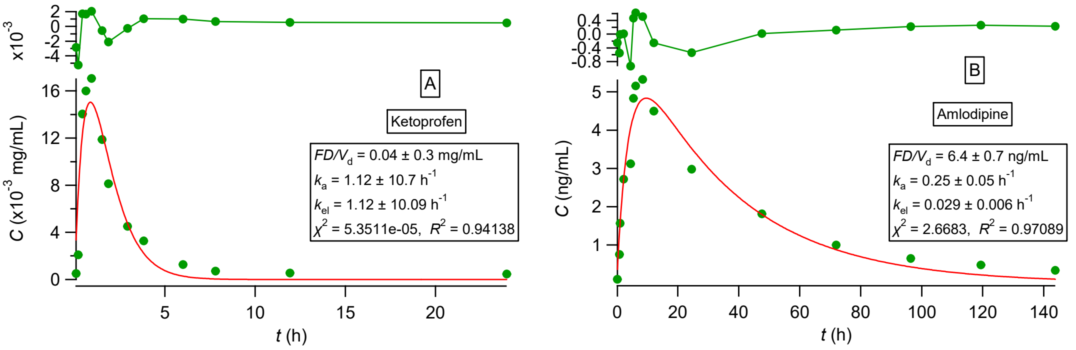

Figure S1. Fittings of Bateman Equation (Eq.2). (A)Ketoprofen experimental data [33]; (B) Amlodipine experimental data [34].The top panel depicts the fit residuals.

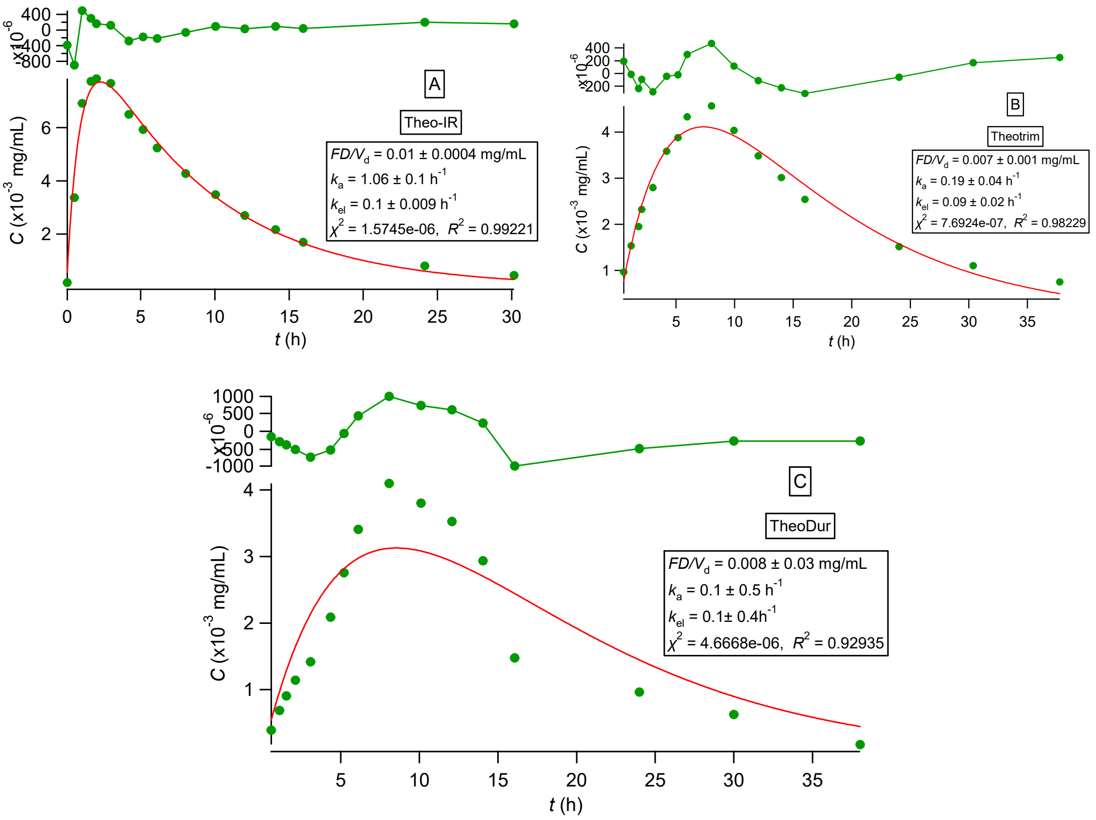

Figure S2. Fittings of Bateman Equation (Eq. 2). (A) Theophylline (Immediate Release) experimental data [35]; (B) Theotrim (Extended Release) experimental data [35]; (C) TheoDur (Extended Release) experimental data [35]. The top panel depicts the fit residuals.

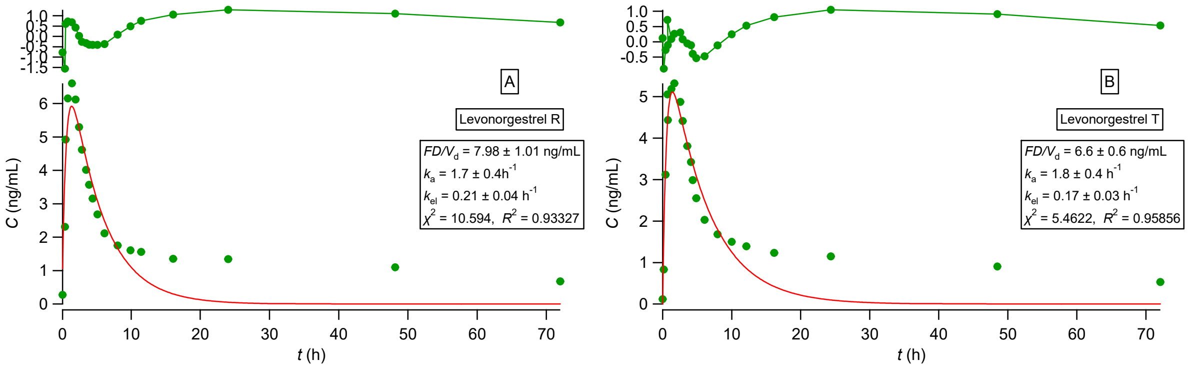

Figure S3. Fittings of Bateman Equation (Eq. 2). (A) Levonorgestrel (Reference) experimental data [36] ;(B) Levonorgestrel (Test) experimental data [36].The top panel depicts the fit residuals.
